# The underlying pathological mechanism of ferroptosis in the development of cardiovascular disease

**DOI:** 10.3389/fcvm.2022.964034

**Published:** 2022-08-08

**Authors:** Li-Li Zhang, Rui-Jie Tang, Yue-Jin Yang

**Affiliations:** ^1^State Key Laboratory of Cardiovascular Disease, Department of Cardiology, Fuwai Hospital, National Center for Cardiovascular Diseases, Chinese Academy of Medical Sciences and Peking Union Medical College, Beijing, China; ^2^Department of Cardiology, The First Affiliated Hospital of Zhengzhou University, Zhengzhou, China

**Keywords:** ferroptosis, cardiovascular disease, mechanism, pathology, metabolism

## Abstract

Cardiovascular diseases (CVDs) have been attracting the attention of academic society for decades. Numerous researchers contributed to figuring out the core mechanisms underlying CVDs. Among those, pathological decompensated cellular loss posed by cell death in different kinds, namely necrosis, apoptosis and necroptosis, was widely regarded to accelerate the pathological development of most heart diseases and deteriorate cardiac function. Recently, apart from programmed cell death revealed previously, ferroptosis, a brand-new cellular death identified by its ferrous-iron-dependent manner, has been demonstrated to govern the occurrence and development of different cardiovascular disorders in many types of research as well. Therefore, clarifying the regulatory function of ferroptosis is conducive to finding out strategies for cardio-protection in different conditions and improving the prognosis of CVDs. Here, molecular mechanisms concerned are summarized systematically and categorized to depict the regulatory network of ferroptosis and point out potential therapeutic targets for diverse cardiovascular disorders.

## Introduction

Cardiovascular diseases (CVDs) mean an array of diseases, including atherosclerosis, hypertension, acute coronary syndrome, heart failure (HF), cardiomyopathy, arrhythmia, *etc.*, as the major cause of morbidity and mortality in the global population, imposing heavy economic pressure on families and community. Therefore, to prevent the occurrence and development of CVDs, its pathological mechanisms have become the focus of medical research in recent years.

Iron is an essential microelement for multiple life processes, and the imbalance of iron homeostasis can cause many adverse consequences. Iron overload is associated with iron deposition in various tissues including the heart, in 1981, Sullivan et al. formally proposed the hypothesis of iron-derived heart disease (1). Since then, iron overload was shown to play a pivotal role in the pathogenesis of CVDs and enhance the risk of cardiovascular morbidity (2). Iron overload can induce a novel iron-dependent form of cell death that is neither apoptosis nor necrosis, called ferroptosis (3). Cell death results in the onset and deterioration of acute or chronic disorders, often accompanied by dysregulation of inflammation, cellular dysfunction, and tissue damage. Acute cardiomyocytes (CM) death can cause myocardial infarction (MI) and ischemia-reperfusion (I/R) injury, while chronic progressive CMs death can cause compensatory hypertrophy, eventually leading to HF or cardiomyopathy (4). Ferroptosis is distinguished from other forms of death. And its main feature is the oxidative modification of phospholipid membranes by iron-dependent mechanisms, coupled with a substantial buildup of lipid peroxides. The pathological role of ferroptosis in atherosclerosis, I/R injury, cardiomyopathy, HF and other CVDs has been widely reported, and targeted intervention of ferroptosis effectively prevented the occurrence and development of these diseases (5–9). This review focuses on the underlying pathological mechanism of ferroptosis in CVDs, regarding oxidative stress, inflammation, metabolic disorders and mitochondrial damage, and provides targeted evidence for treatment.

## Characteristics of ferroptosis

Dixon et al. (3) showed that the selectively RAS-lethal small molecule erastin not only modulated the mitochondrial voltage-dependent anion channel (VDAC), but also inhibited the function of SLC7A11, an important subunit of the cystine/glutamate reverse transporter system Xc^–^ (xCT), reducing intracellular glutathione (GSH) synthesis, further increasing the accumulation of iron-dependent lipid peroxide and inducing the death of RAS mutant fibrosarcoma cell lines. They formally named this new form of regulated cell death triggered by erastin as ferroptosis: an iron-dependent non-apoptotic form of cell death caused by the imbalance of intracellular synthesis and degradation of ROS and lipid peroxides (3). Different from typical cell death form, ferroptosis cannot be inhibited by inhibitors of apoptosis and pyroptosis, but can be inhibited by iron chelators (e.g., deferoxamine), antioxidants (e.g., tea polyphenol) (10, 11). Moreover, ferroptosis has unique morphological features and biochemical markers. Morphologically, there was no cell shrinkage, chromatin condensation, formation of apoptotic bodies, or disintegration of the cytoskeleton, but significant shrinkage of mitochondria and reduction of mitochondrial cristae were observed (3). Regarding distinguishing biomarkers, they are associated with the accumulation of intracellular iron ions and ROS accompanied by the decrease in GSH metabolism, as exemplified in Table 1.

**TABLE 1:** Major regulatory indicators and potential markers of ferroptosis.

Characteristics	Indicators	Key biomarkers
Iron accumulation	Total iron or ferrous ion	Tf, TfR, ferritin, iron-sulfur protein, NCOA4, etc.
Lipid peroxidation	ROS, increased oxidized polyunsaturated fatty acids	NQO1, NOX1, SOD, MDA, NRF2, Arachidonoyl-CoA, PTGS2/COX-2, LOX, ACOT, ACSL, etc.
Decreased GSH metabolism	Ratio of GSH to GSSG	GPX4, GSH, xCT, SLC7A11, SLC3A2, p53, etc.
Mitochondrial dysfunction	Measurement of mitochondrial membrane potential and respiratory function, mtDNA damage	VDAC1, VDAC2, MPTP, ETC complexes, etc.

Tf, transferrin; TfR, transferrin receptor; NCOA4, nuclear receptor coactivator 4; MDA, malondialdehyde; ROS, reactive oxygen species; NQO1, NAD(P)H: quinone oxidoreductase 1; NOX, NADPH oxidase; SOD, superoxide dismutase; NRF2, nuclear factor erythroid 2-related factor 2; GPX4, glutathione peroxidase 4; GSH, glutathione; GSSG, oxidized glutathione; xCT, system Xc-; SLC7A11, solute carrier family 7 member 11; SLC3A2, solute carrier family 3 member 2; VDAC, voltage-dependent anion channel; PTGS2, Prostaglandin G/H synthase-2; COX, cyclooxygenase; LOX, lipoxygenase; ACOT, acyl-CoA thioesterase; ACSL, acyl-CoA synthetases; MPTP, mitochondrial permeablity transition pore; ETC, electron transfer chain.

## Mechanisms of ferroptosis

Iron overload is a prerequisite for ferroptosis, and various iron chelators can inhibit ferroptosis. Transferrin receptor 1 (TfR1) can promote the transfer of extracellular iron into cells, and the silencing of its encoding gene prevented ferroptosis caused by erastin. Iron supplementation accelerated erastin-induced ferroptosis (12). Treatment of dopaminergic cells with ferric ammonium citrate to mimic the iron overload showed that ferroptosis occurred before apoptosis and could be rescued by ferroptosis inhibitors (13). These results further demonstrated the necessity of iron overload in the process of ferroptosis.

The accumulation of lipid peroxide is another crucial element in the execution of ferroptosis. Cellular or organelle membranes are especially vulnerable to attack by ROS due to their high polyunsaturated fatty acids, called lipid peroxidation. An important source of ROS in mammalian cells is mitochondria. Among mitochondrial metabolic activities, tricarboxylic acid (TCA) cycle starts with acetyl-CoA from glucose or fatty acid metabolism, and transfers electrons to the electron transport chain (ETC), completing oxidative phosphorylation (OXPHOS). Once the electrons are leaked from ETC complexes I and III, superoxide will be produced and converted to H_2_O_2_ by superoxide dismutase (SOD) (14). Subsequently, Fe^2+^ released via divalent metal transporter 1 (DMT1) can react with H_2_O_2_ to yield hydroxyl (⋅OH) or alkoxy (RO⋅) radicals, known as “Fenton reaction,” resulting in the production of reactive poisonous aldehydes, in the form of 4-hydroxynonenal (4-HNE) and malondialdehyde (MDA) (15–17). In addition, ROS can induce autophagy to accelerate the degradation of ferritin and enhance the expression of TfR1 leading to ferroptosis (18). In turn, ferroptosis can also significantly increase ROS production, disrupt mitochondrial membrane potential, and promote mitochondrial fission (19). Accordingly, mitochondrial damage and energy metabolism disorders exacerbate the vicious cycle between lipid peroxidation and ferroptosis.

As confirmed by Dixon et al., xCT-mediated cystine absorption from extracellular space accompanied by the export of intracellular glutamate could regulate ferroptosis (3). In the cytoplasm, cystine turns into cysteine, a main raw material of GSH synthesis. GSH produced above serves as an electron donor to reduce toxic lipid peroxides into non-toxic alcohols under the enzymolysis of glutathione peroxidase 4 (GPX4) (20). Hence, the xCT-GSH-GPX4 axis conducts the dominant defensive mechanism against cellular ferroptosis. Upregulation of p53 significantly reduced the expression of SLC7A11, which inhibited xCT mediated-cystine uptake and resulted in ferroptosis (21). RSL3, erastin and sulfasalazine are direct targets of GPX4 and make cancer cells more sensitive to ferroptosis (22). It can be inferred that any methods causing xCT inhibition, GSH or GPX4 depletion might promote ferroptosis. In addition, ferroptosis suppressor protein 1 (FSP1), formerly known as apoptosis-inducing factor mitochondria-associated 2, protects cells from GPX4 deletion-induced ferroptosis (23). Treatment of lung cancer cells with iFSP1, an inhibitor of FSP1, potentiated ferroptosis (24). Therefore, antagonism of FSP1 can lead to the occurrence of ferroptosis.

Moreover, ferroptosis as a type of necrotic cell death is mostly accompanied by inflammatory manifestations. Ferroptosis-related necroinflammation was observed in mice with acute kidney injury (25). Ferroptotic cells can release damage-associated molecular patterns (DAMPs) to activate immune cells, which further amplify ferroptosis by releasing inflammatory mediators such as interleukin (IL)-1β and tumor necrosis factor (TNF)-α. In addition, during ferroptosis, polyunsaturated fatty acids, especially arachidonic acids, are prone to peroxidation. The lipoxygenase (LOX) and cyclooxygenase (COX)-generated metabolites of arachidonic acid (hydroxyeicosatetraenoic acid, leukotriene, and prostaglandins) are also involved in inflammatory response (26). The above shows that ferroptosis is closely related to inflammation and immunity, and the persistence of inflammation may form a self-amplifying circuit of ferroptosis.

In sum, oxidative stress, inflammation, metabolic disorders, and mitochondrial damage are the main cellular and molecular pathological mechanisms in ferroptosis.

## Pathological mechanism of ferroptosis in cardiovascular diseases

### Oxidative stress

**FIGURE 1:**
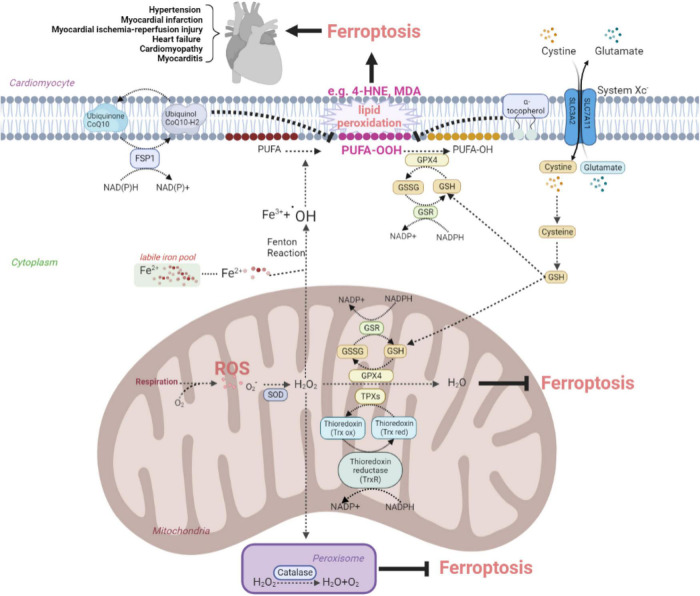
The anti-oxidative system inhibits ferroptosis to protect against cardiovascular disease by eliminating oxidative stress. The figure shows the protective effects of the SOD family, catalase, Trx recycles, system Xc^–^-GSH-GPX4 pathway, FSP1-CoQ10-NAD(P)H pathway, and tocopherol on the elimination of ROS and reducing the conversion to lipid peroxide, such as toxic MDA and 4-HNE. ROS, reactive oxygen species; SOD, superoxide dismutase; FSP, ferroptosis suppressor protein; CoQ, coenzyme Q; GSH, glutathione; GSSG, oxidized glutathione; GSR, glutathione reductase; GPX, glutathione peroxidase; 4-HNE, 4-hydroxynonenal; MDA, malondialdehyde; TPX, thioredoxin peroxidase; Trx red, reduced thioredoxin; Trx ox, oxidized thioredoxin; PUFA, polyunsaturated fatty acid.

Oxidative stress is involved in assorted CVDs like MI, I/R injury, and HF. Excessive production of ROS impairs cellular lipids, resulting in ferroptosis. In the cardiovascular system, the antioxidant system controls intracellular ROS and interacts with biological components to maintain redox equilibrium. This system contains counteroxidant enzymes such as SOD, catalase, GPX, and thioredoxin (Trx), and non-enzymatic antioxidants such as tocopherol and coenzyme Q10 (CoQ10) are shown in Figure 1. This system eliminates ROS by chelating metal ions, enhances the production of endogenous antioxidants, and defends against ferroptosis in the cardiac tissues (27–30).

#### Superoxide dismutase system

Three isoforms of SOD system in mammals are SOD1, SOD2, and SOD3, as the first line of defense against oxygen-derived free radicals in the cardiovascular system. Under the exposure to oxidative stress, they can be rapidly activated to catalyze superoxides into oxygen and H_2_O_2_. Among three members of SOD family, SOD1, an isoform located in the cytoplasm containing Cu^2+^ and Zn^2+^, outweighs SOD2 and SOD3 in the expression of abundance. A cohort study showed that SOD1 allelic mutations in the population were linked with an elevated risk of death from cardiovascular complications (sudden death, fatal MI, or stroke) (31). SOD1 deletion promoted the occurrence of ferroptosis (32). Sustained delivery of SOD1 could improve myocardial I/R injury (33). SOD2 is a manganese-containing isoenzyme found in mitochondria, also associated with ferroptosis (34). Deficiency of SOD2 increases ROS in CMs, leading to subsequent overproduction of 4-HNE inside mitochondria and impairment of mitochondrial bioenergy production, which is one of the causes of lethal dilated cardiomyopathy (35). The structure of SOD3 is similar to SOD1, and upregulation of SOD3 is conducive to rid the vascular system of oxidative damage, alleviating the severity of hypertension and coronary arteriosclerosis (36). Therefore, the SOD system may protect from the occurrence of ferroptosis in CVDs by reducing superoxides.

#### Catalase

Catalase located in the peroxisome varies in different developmental stages of organisms, which is manipulated by peroxisome proliferator-activated receptors (PPARs) and decomposes H_2_O_2_ into H_2_O and oxygen (37). Upregulation of catalase has been reported to increase ferroptosis resistance in xCT-deficient mouse blast fibroblasts. Besides, catalase activation is cardioprotective to H_2_O_2_-induced stress in myocardial I/R injury by alleviating ferroptosis (38, 39).

#### Glutathione peroxidase system

Glutathione peroxidase 4 family contain eight members, namely GPX1–GPX8. The similar denominator among diverse GPXs gifts their abilities to reduce H_2_O_2_ and hydroperoxides to H_2_O. GPX4 is unique in the GPXs family which can reduce phospholipid hydroperoxides of membranes using redox equivalents from GSH. A proteomic analysis showed that GPX4 downregulation using specific siRNA or chemical inhibitor RSL3 caused CMs ferroptosis during MI (40). Activation of nuclear factor erythroid 2-related factor 2/xCT/GPX4 signaling pathway attenuates CMs ferroptosis in doxorubicin-induced cardiomyopathy and myocardial I/R injury by inhibiting oxidative stress (41, 42). Besides, the ferroptosis inhibitor ferrostatin-1 markedly prevented pathological myocardial remodeling and fibrosis in angiotensin II-induced hypertensive cardiomyopathy by attenuating the upregulation of ferrous ion levels and lipid peroxidation in mouse microvascular endothelial cells (ECs) through modulating the xCT/GPX4 signaling (43). The Chinese herbal medicine, Tongxinluo, prevented atherosclerosis, and the xanthohumol reduced myocardial I/R injury as well, both through modulating GPX4 expression to decrease ROS (44, 45). Thus, chemicals targeting GPX4 signaling pathway could act as potential drugs to various CVDs by suppressing ferroptosis.

#### Thioredoxin system

Thioredoxin reductase (TrxR), Trx and NADPH together constitute the Trx system. TrxR is an NADPH-dependent dimeric selenozyme required for reducing and recycling the oxidized Trx1. TrxR was verified to reduce cell sensitivity to erastin (46). Cardiac-specific TrxR knockout (KO) aged mice were more prone to dilated cardiomyopathy due to increased ROS and dysregulated mitochondrial energy metabolism (47). Additionally, Trx-1 as a small molecule protein participates in redox reactions. Overexpression of Trx-1 could inhibit ferroptosis by increasing GPX4 and GSH (48), which indicated the GSH-Trx cross-talk ensured cell survival. In Trx1 KO mice, increased oxidation of the mammalian target of the rapamycin (mTOR) signaling leads to impaired phosphorylation of substrates and mitochondrial respiratory dysfunction, manifesting as systolic dysfunction in HF (49). The Trx system delivers electrons for thioredoxin peroxidase (TPX), whose function is similar to the GPX to remove ROS by reducing H_2_O_2_ and hydroperoxides at a rapid reaction rate (50). Thus, it is speculated that the Trx system alleviates ferroptosis to exert cardioprotective effects by modulating the reduction/oxidative balance.

#### Tocopherol

Vitamin E has eight isomers that inhibit lipid peroxidation by donating phenolic hydrogen to peroxyl radicals, producing inactive tocopherol radicals. Alpha-tocopherol was the most prevalent and physiologically type of vitamin E (51). *In vitro*, alpha-tocopherol was demonstrated to rescue GPX4-deficient cells from ferroptosis, indicating that GPX4 and vitamin E synergistically maintain lipid redox equilibrium (52). Alpha-tocopherol may decrease myeloperoxidase expression, reducing oxidative and inflammatory reactions induced by I/R damage in mice, ultimately, preserving heart function (53). However, Keith et al. conducted a clinical trial showed that vitamin E supplementation significantly elevated alpha-tocopherol plasma concentrations in the treated group but failed to affect any other markers of oxidative stress and prognosis or function in class III or IV advanced HF (54). It is possible that alpha-tocopherol supplementation alone is not sufficient to combat severe oxidative stress and may achieve the desired therapeutic effect in combination with other natural antioxidants.

#### CoQ10 system

CoQ10 is named because of the mammalian CoQ containing 10 isoprene units. CoQ10 mainly exists in two forms, namely ubiquinone (oxidized form) and ubiquinol (reduced form). Ubiquinone can be transformed into ubiquinol by losing two electrons, and ubiquinol plays the main anti-oxidant role. FSP1 participates in the regulation of CoQ10 antioxidant system to alleviate ferroptosis, in a GSH- and GPX4-independent manner. The N-terminal end of FSP1 containing a typical myristoylation motif can bind to the phospholipid bilayer of cell membrane. The reduced form, ubiquinol, converts to the oxidative form, ubiquinone, after chemically reacting with lipid peroxide radicals released from the metabolism of membrane lipid, and eventually shut down the ferroptosis. Furthermore, under the catalysis of FSP1, ubiquinone can be reduced to ubiquinol in the cell membrane using NAD(P)H (23). Recently, an *in vivo* experiment has proven that FSP1 upregulation alleviates myocardial injury induced by septicemia through inhibition of ferroptosis (54). In short, the FSP1-CoQ10-NAD(P)H axis underlies another indispensable mechanism for myocardium protection under CVDs.

### Inflammation

#### Endogenous damage-associated molecular patterns

**FIGURE 2:**
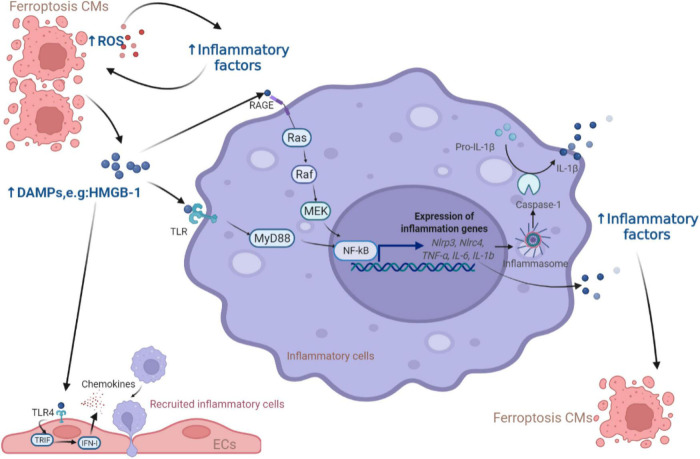
The role of DAMPs released from ferroptotic CMs in inflammation. The release of DAMPs from ferroptotic cells activates the downstream NF-κB inflammatory pathway through the TLR and RAGE receptors of inflammatory cells, leading to the production of inflammatory factors (e.g., NF-κB, IL-6, IFN-γ, TNF-α, and IL-1β) which were also produced due to the increase of ROS to induce more CMs ferroptosis. In addition, ECs respond to DAMPs via TLR4 receptors and release chemokines to promote more inflammatory cells entering the myocardial microenvironment. CM, cardiomyocyte; ROS, reactive oxygen species; DAMP, damage-associated molecular pattern; HMGB, high mobility group box; RAGE, receptor for advanced glycation end products; TLR, Toll-like receptors; MEK, mitogen-activated protein kinase kinase; NF-κB, nuclear factor-kappa B; NLRP, NOD-like receptor thermal protein domain associated protein; NLRC, NLR family CARD domain containing; MyD88, myeloid differentiation factor 88; IL, interleukin; TRIF, TIR-domain-containing adapter-inducing interferon-β; IFN, interferon; EC, endothelial cell.

Damaged or dead CMs can generate or release host-derived danger signaling molecules DAMPs, which have been identified as endogenous dangerous signals for the innate immune system (55). The high mobility group box 1 (HMGB1) protein is a type of DAMP that can be released by ferroptotic cells. Ferroptosis inducers, erastin, sorafenib and RSL3, all trigger the release of HMGB1, which recruits pro-inflammatory macrophages and microglia via activating receptors for advanced glycation end products (RAGE)-nuclear factor-kappa B (NF-κB) pathway. Upregulation of HMGB1 expression has been shown to contribute to chronic HF and ischemic heart disease (56–59). Besides, inflammasomes are multi-protein complexes involving intracytoplasmic pattern recognition receptors. Inflammasomes were activated by DAMPs, followed by activating pro-inflammatory proteases and producing the corresponding mature cytokines (60, 61). Englestrin, a selective sodium-glucose cotransporter 2 inhibitor, significantly improved cardiac function in adriamycin cardiomyopathy by attenuating CMs ferroptosis through inhibition of the NOD-like receptor thermal protein domain associated protein 3 (NLRP3) inflammasomes related pathways (62). DAMPs are also processed by antigen-presenting cells like dendritic cells (DCs) through Toll-like receptors (TLRs) and presented to T cells to initiate an immune response. Besides, DAMPs released from ferroptotic ECs facilitates neutrophil adhesion to coronary vessels via activating the TLR4/TIR-domain-containing adapter-inducing interferon-β (TRIF)/interferon I (IFN I) pathway, resulting in neutrophil recruitment to the damaged myocardium (63). These discoveries shed light on the contribution of the immune cells activated by DAMPs from ferroptotic cells leading to CVDs, as summarized in Figure 2.

#### Pro-inflammatory factors

Inflammation is tightly associated with oxidative stress, which triggers a range of pro-inflammatory factors such as NF-κB, IL-6, IFN-γ, TNF-α (64–67). Besides, studies have confirmed that the NF-κB pathway and IL-6/JAK2/STAT3 pathway activation could induce ferroptosis (68, 69). IFN-γ could down-regulate the expression of two subunits of xCT, SLC3A2 and SLC7A11, through the ASK1/JNK or JAK1-2/STAT1 pathway, reducing cellular uptake of cystine and thus promoting ferroptosis (70, 71). In cardiovascular-related diseases, the inhibition of these inflammatory pathways could rescue the ferroptotic CMs or ECs. For example, the ferroptosis inhibitor ferrostatin-1 improved sepsis-induced cardiac systolic dysfunction partly through alleviating the TLR4/NF-κB signaling pathway and reducing TNF-α and IL-6 (72). A hormone elabela significantly attenuated ferroptosis in hypertensive mice by inhibiting the cardiac IL-6/STAT3 signaling pathway (43). Nevertheless, the involvement of IFN-γ in the occurrence of ferroptosis has not been investigated in CVDs and needs to be further studied.

Furthermore, membrane-bound arachidonic acid was mainly found in the phospholipids of cell membranes and subject to COX, LOX and cytochrome P450 to yield a series of inflammatory mediators, like prostaglandins and leukotrienes, causing aseptic inflammation (73, 74). Reducing intracellular levels of arachidonoyl in CMs blocked neutrophil replenishment and prevented the onset of ferroptosis. Eliminating oxidative productions from arachidonic acid could also inhibit NF-κB pathway activation (75). Thus, agents against metabolites of arachidonic acid can play an anti-inflammatory or cytoprotective role in CVDs caused by ferroptosis.

#### Immune cells

**FIGURE 3:**
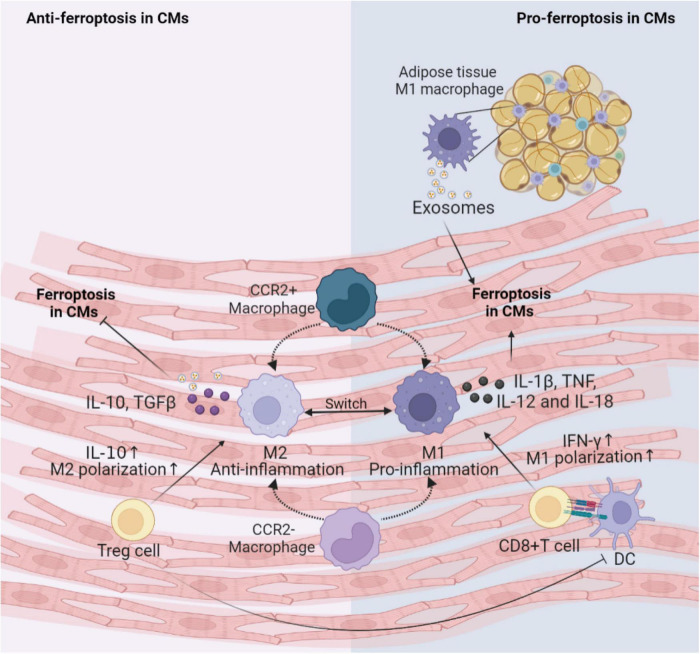
Immune cells involved in the regulation of CMs ferroptosis. This figure summarizes macrophages and T cells activity regulating the ferroptosis following cardiac injury. The cross-talk between these immune cells and CMs depends on various inflammatory factors and exosomes. DC, dendritic cell; CM, cardiomyocyte; IL, interleukin; TNF, tumor necrosis factor; IFN, interferon; TGF, transforming growth factor; CCR, C-C motif chemokine receptor 2.

During myocardial injury, different types of immune cells interact with each other, and with CMs, fibroblasts, and ECs to elicit inflammatory responses. Among these immune cells, the macrophages and T cells are the two most studied types in the cardiovascular field. Here, we focus on the possible effects of these two types of cells on ferroptosis in CVDs, shown in Figure 3.

Generally, the first immune cell type to respond to myocardial injury is the tissue-resident macrophages. Tissue-resident C-C motif chemokine receptor 2 (CCR2)^–^ macrophages originated from the embryonic developmental stage are distinguished from tissue-resident CCR2^+^ macrophage repopulated by monocytes extravasating into the myocardium. Unlike CCR2^+^ macrophages, tissue-resident CCR2^–^ macrophages could inhibit monocyte recruitment (76). Apart from preventing fibrosis and stimulating angiogenesis (77), CCR2^–^ macrophages also improve cardiac repair via promoting clearance of apoptotic CMs, up-regulating anti-inflammatory mediators IL-10 and TGF-β, and down-regulating pro-inflammatory mediators IL-1β, TNF-α, IL-6 and IFN-γ after MI (78). Consumption of resident CCR2^–^ macrophages leads to activation of inflammasomes in CMs, abnormal mitochondrial accumulation, and postinfarction ventricular dysfunction (79). It can be inferred that cardiac resident CCR2^–^ macrophages may engulf CMs undergoing ferroptosis and reduce the inflammation to inhibit ferroptosis. Subsequently, the circulating CCR2^+^ mononuclear macrophages infiltrate into the myocardial injury environment in greater number than the CCR2^–^ macrophages and polarize to M1 pro-inflammatory phenotype and M2 reparative phenotype (80). Iron overload promotes polarization of M1 macrophages (81). M1 macrophages are more resistant than M2 macrophages to ferroptosis due to high intracellular levels of inducible nitric oxide synthase and nitric oxide radicals which inhibit lipid peroxidation (82, 83). M1 macrophages secrete large amounts of pro-inflammatory factors such as IL-1β, TNF, IL-12, and IL-18, increase ROS and have the potential to induce ferroptosis in adventitial hypoxic CMs (84, 85). Besides, exosomes derived from M1 macrophages in obese adipose tissue carry miR-140-5p to induce ferroptosis in CMs by targeting xCT to inhibit GSH synthesis, leading to abnormal left ventricular contraction in obese mice (86). While it has been shown that M2 macrophage-derived exosomes inhibit cell ferroptosis (87). Hence, M1 macrophages may be a key inducer of ferroptosis in the microenvironment of myocardial inflammation. Future studies are needed to prove the effects of macrophages on the onset of ferroptosis in CMs.

Infiltrating T cells activated by dendritic cells (DCs) may also regulate the occurrence of ferroptosis in CVDs. Cytotoxic CD8^+^ T cells can lead to poor cardiac remodeling after ischemia, acute hypertensive heart injury, acute rejection of allogeneic heart retransplantation and other CVDs, possibly by inducing CMs ferroptosis through releasing IFN-γ and promoting M1 polarization (88). For CD4^+^ effector T cells, the effects of different subtypes are pleiotropic. T helper (Th)1 cells could activate cardiac fibroblasts to induce fibrosis; while regulatory Treg cells could reduce the number of myofibroblasts through inhibiting inflammation via anti-inflammatory cytokine IL-10 in hypertension and HF (89–91). Additionally, Treg cells beneficially influenced wound healing after MI by regulating the polarization of M2 macrophages and inhibiting proinflammatory cytokines production (92). Paracrine action of Treg cells also promoted fetal and maternal CMs proliferation to improve the prognosis of MI during pregnancy (93). Thus, Treg cells may alleviate cellular ferroptosis in the myocardial microenvironment by interacting with CMs and inflammatory cells.

### Metabolic disorders

#### Glucose metabolism disorder

**FIGURE 4:**
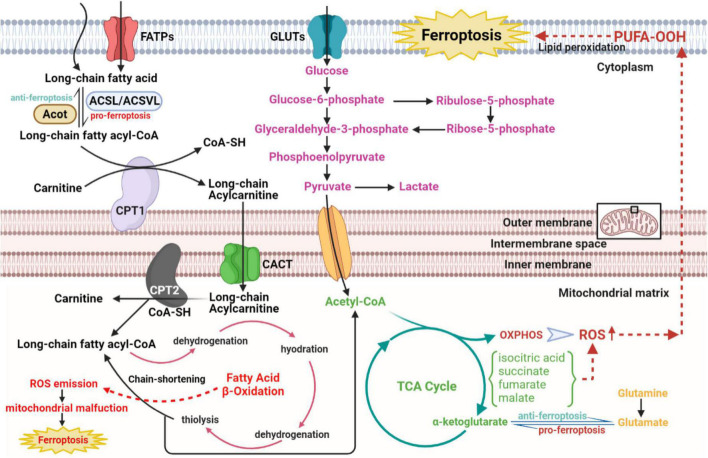
The role of glucose and fatty acid metabolic disorders in the regulatory network of ferroptosis of CVDs. The long-chain fatty acid is activated and transformed into long-chain fatty acyl-CoA with the catalytic activity of ACSL/ACSVL. The long-chain fatty acyl-CoA combines with carnitine and enters fatty acid β-oxidation in the mitochondrial matrix with help of CPT1, CPT2, and CACT. Then acetyl-CoA from the β-oxidation cycle, anaerobic glycolysis, and glutaminolysis gather to strengthen the metabolism of the TCA cycle in mitochondrial matrix, which activates downstream OXPHOS and ROS, eventually resulting in cellular ferroptosis. CVDs, cardiovascular diseases; ACSL, long-chain acyl-CoA synthases; ACSVL, very long-chain acyl-CoA synthases; CoA, coenzyme A; CPT1, carnitine palmitoyltransferase1; CPT2, carnitine palmitoyltransferase2; CACT, carnitine-acylcarnitine translocase; OXPHOS, oxidative phosphorylation; ROS, reactive oxygen species.

As the Figure 4 shows that under aerobic circumstances, glucose is initially transformed into pyruvate through the glycolytic pathway and subsequently undergoes the TCA cycle and mitochondrial OXPHOS processes to complete metabolism (94, 95). At the initial stage of glycolysis, the glucose molecule is phosphorylated by hexokinase to produce glucose 6-phosphate, which is then further converted to fructose 6-phosphate by glucose 6-phosphate isomerase, to subsequently produce fructose 1,6-diphosphate catalyzed by fructokinase 6-phosphate kinase 1, the main rate-limiting enzyme in the glycolytic process. Simultaneously, fructose 6-phosphate is bypassed by glucose-6-phosphate dehydrogenase and enter the pentose phosphate pathway to produce the essential biosynthetic precursor and NADPH, 60% of which required in animals is produced in this manner. NADPH participates in multiple anabolic reactions, responsible for the recycling of GSH, ubiquinone and Trx, etc., which is crucial for ensuring the redox homeostasis in the body (96). Pyruvate produced by glycolysis enters the mitochondria to generate acetyl-CoA, which enters the TCA cycle to generate NADH, FADH_2_ coupled with OXPHOS. Mitochondrial OXPHOS is accompanied by the transfer of electrons in the ETC to molecular oxygen, leading to the generation of ROS. Therefore, it can be speculated that glucose metabolism in CMs is closely linked to ferroptosis. Echoing the above hypothesis, appropriate blockade of glycolysis or TCA significantly inhibited ferroptosis induced by erastin, cystine depletion or RSL3 treatment, as manifested by stabilizing mitochondrial membrane potential and decreasing lipid peroxides accumulation (97, 98). According to the metabolomic analysis, CMs exposed to the ferroptosis inducer RSL3 showed markedly elevated isocitric acid and ketoglutarate (KG) contents (99). Accumulation of some TCA circulating metabolites, alpha-KG, succinate, fumarate and malate, can all contribute to lipid ROS accumulation and ferroptosis. Additionally, alpha-KG dehydrogenase/succinate/aconitase activity was enhanced during the onset of ferroptosis due to cysteine deprivation (97, 100). In the TCA cycle, glutamine can be catabolized to glutamate by glutaminase, which is further metabolized to alpha-KG for replenishment. The glutamine-fueled intracellular metabolic pathway is required for ferroptosis induced by cysteine deprivation (101). Inhibition of glutamine catabolism reduces cardiac injury triggered by I/R, offering a prospective therapy (102). These results suggest that glucose metabolism products enhance the mitochondrial ROS generation, further amplifying the ferroptosis signal.

#### Fatty acid metabolism disorder

Given that fatty acids are the core energy source for the myocardium, concerns about lipid metabolism have crucial implications in ferroptosis from CVDs (Figure 4). According to the basic perspectives on fatty acid metabolism, three stages are involved in this process, named activation, translocation and β-oxidation. Some biomolecule evolved in the above three stages, either metabolites or relative enzymes, can spark cellular ferroptosis. As the first reaction of fatty acid catabolism, long-chain acyl-CoA synthetases (ACSLs) and very long-chain acyl-CoA synthetases (ACSVLs) converts different kinds of long-chain fatty acid to corresponding fatty acyl-CoA. Among these enzymes, ACSL4 mostly activates the long-chain fatty acids and subsequently enriches cellular membrane with polyunsaturated ones. Specifically, two long-chain fatty acids, arachidonic acid, and adrenoic acid, can be both converted to arachidonoyl-CoA and adrenoyl-CoA via ACSL4 and coenzyme A. Following the activation of long-chain fatty acids, arachidonoyl-CoA and adrenoyl-CoA are used to synthesize phosphatidylethanolamine and phosphatidylinositol, composing negatively charged cell membrane. Phosphatidylethanolamine can often be oxidized, triggering the ferroptosis of cells, particularly under the treatment with RSL3 (103). Consistent with the above findings, ACSL4, iron and MDA level in reperfusion myocardium gradually rose with the prolongation of myocardial ischemic time (104). Serving as the functionally well-matched adversary to ACSLs, Acyl-CoA thioesterases (ACOTs) accelerate fatty acid synthesis with the substrate fatty acyl-CoA, and the balance between ACSLs and ACOTs governs the dynamic equilibrium of fatty acid metabolism. As reported in cardiovascular basic researches, ACOT1, an isoform of ACOTs working in cytoplasm, hydrolyzes fatty acyl-CoAs, varying from short-chain ones to very long-chain ones, to free fatty acids and conenzyme A in myocardium (105, 106). Under the condition of doxorubicin-induced cardiomyopathy, Acot1 knockdown sensitizes CMs to ferroptosis, whereas ACOT1 overexpression poses defensive effect on cellular ferroptosis. This beneficial effect is conducted through ACOTs’ function of lipid components alteration and acyl-CoA hydrolysis, evidenced by increased anti-oxidative ω-3 polyunsaturated fatty acids and docosahexaenoic acid abundance in the hearts of ACOT1-overexpressed mice (107). Meanwhile, in the stage of fatty acid activation, the adipokine chemerin deriving from adipose tissue inhibits fatty acid oxidation and maintains fatty acid levels to confer ferroptosis resistance. Chemerin inhibition shuts down the anti-oxidative CoQ system, followed by ROS explosion, additionally, its deficiency remarkably attenuates glycerophospholipid species accumulation, including phosphatidic acid, phosphatidylcholine, phosphatidylglycerol and intact sphingolipids, with an immediate increase in the replenishment of their corresponding oxidative and catabolic products (108, 109). Subsequent to the translocation stage, the substrate, fatty acyl-CoA, enters the β-oxidation cycle in mitochondrial matrix. After every four steps of β-oxidation, the carbon chain of acyl-CoA is shortened by two carbons, with acetyl-CoA released. Then, explosive acetyl-CoA production from fatty acid β-oxidation accelerates downstream TCA cycle and excites ROS-induced ferroptosis. Hence, ideal component remodeling and maintenance of free fatty acids metabolic balance are predicted to improve the sensitivity of CMs to ferroptosis.

#### Iron metabolism disorder

The balance of iron homeostasis in the body depends on the metabolic regulation of iron absorption, recycling and release. The absorption of dietary iron into the blood is regulated by DMT1 of intestinal epithelial cells. Iron recycling is obtained from senescent red blood cells phagocytosed by reticuloendothelial macrophages. Ferroportin (FP) is the only known mammalian iron exporter capable of releasing iron into the circulation. Hepcidin, a liver-derived hormone, can degrade FP and reduce the release of iron to the circulation. Therefore, in addition to nutritional iron deficiency, the disorders of the process mentioned above such as FP mutation and abnormal hepcidin levels (affected by inflammation, hypoxia, hemochromatosis protein, TfR, and other factors) can lead to the imbalance of iron homeostasis in the body (110).

**FIGURE 5:**
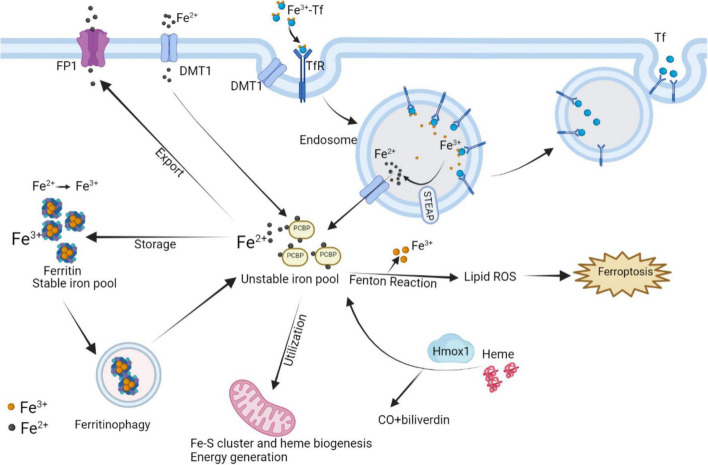
Interaction of intracellular iron metabolism and ferroptosis. Fe^3+^ and Tf bind to form Fe^3+^-Tf complex and enter the cell by means of TfR1 via endocytosis. In endosomes, Fe^3+^ is liberated from Tf and catalyzed by STEAP to Fe^2+^, while Tf is released extracellularly by exocytosis. The process Fe^2+^ released from endosomes or absorbed into the cells depends on DMT1. In mitochondria, the utilization of Fe^2+^ is mainly the synthesis of Fe–S clusters and heme, which can be degraded by Hmox1 to regenerate Fe^2+^. Excess Fe^2+^ can store in ferritin by converting into Fe^3+^, while ferritinophagy can re-release free iron. Intracellular Fe^2+^ can also be exported through FP1. The above processes participate in the regulation of intracellular Fe^2+^ content to control Fenton reaction, thereby regulating the occurrence of ferroptosis. FP1, ferroportin1; DMT1, divalent metal transporter 1; Tf, transferrin; TfR1, transferrin receptor 1; Hmox1, Heme oxygenase 1; STEAP3, six-transmembrane epithelial antigen of the prostate 3.

At the level of individual CMs, the metabolic mechanisms maintaining iron homeostasis are mainly illustrated in Figure 5, associated with CMs ferroptosis. The uptake and transport of iron in the CMs mainly depend on TfR1, which takes in the Fe^3+^-transferrin (Tf) complex into the cytoplasm via endocytosis, and finally Fe^3+^ mainly stores in the form of ferritin and hemosiderin. Ferritin controls the distribution and storage of iron in cells, preventing iron-catalyzed Fenton reaction and free radical production. Ferritin consists of 24 subunits, including ferritin light chain (Ftl), and ferritin heavy chain (Fth), the latter with ferrous oxidase activity can oxidize poly(C)-binding protein (PCBP)-carried Fe^2+^ to Fe^3+^. In ferritin knock-out mice, ferroptosis induced by decreased xCT expression in CMs predisposed the mice to cardiomyopathy (9). BTB and CNC Homology 1 (BACH1) is a heme-binding transcription factor required for the appropriate regulation of oxidative stress responses and metabolic pathways associated with heme and iron. BACH1 inhibited Fth1 and Ftl1 encoding ferritin, exacerbating MI by promoting ferroptosis (111). Degradation of ferritin, especially Fth1, through nuclear receptor coactivator 4 (NCOA4) –mediated ferritinophagy resulted in the release of ferritin-bound iron to replenish free Fe^2+^ (112). Inhibition of NCOA4-mediated ferritinophagy contributed to myocardial hypertrophy through mitochondrial iron overload (113). Regarding Fe^2+^, it can be taken up into the CMs by DMT1. Additionally, Fe^2+^ can be transformed by Fe^3+^ deoxygenation under the catalysis of the iron oxide reductase six-transmembrane epithelial antigen of the prostate 3 (STEAP3), followed by releasing from the endosome mediated by DMT1 into the ferroptosis-associated unstable iron pool in the cytoplasm. Furthermore, loss of cardiac iron exporter FP1 rapidly caused an eventual fatal impairment of cardiac function in mice, which is associated with intracellular iron deposition in the myocardium (114). Salvia miltiorrhiza injection decreased cardiac iron deposition and inhibited cardiac oxidation by down-regulating DMT1 and TfR1 expression and up-regulating FP1 protein levels (115). The above evidence suggests that reducing cardiac iron uptake and increasing iron excretion is one of the essential mechanisms for enhancing cardiac function.

Heme oxygenase 1 (Hmox1) is a rate-limiting enzyme in the metabolism of the iron porphyrin compound heme, which is broken down into carbon monoxide, biliverdin, and Fe^2+^. The myocardium is rich in heme for the synthesis of myoglobin, cytochrome, etc., therefore, Hmox1 plays an important role in the regulation of myocardial iron metabolism. Hmox1 mediates ferroptosis induced by the release of Fe^2+^ from heme and is responsible for adriamycin-induced cardiotoxicity or cardiac injury with sickle cell disease in mice (7, 116). While Hmox1 also regulates intracellular oxidation levels by stabilizing hypoxia-inducible factor 1α to prevent ischemia-mediated injury (117). Consequently, excessive up-regulation of Hmox1 is toxic, while moderate up-regulation of Hmox1 may have a positive cytoprotective effect on the myocardium.

### Mitochondrial injury

Apart from serving as the primary organelle for intracellular energy generation, mitochondria also play the central role in utilization, catabolism, and anabolisms for iron. Cellular iron homeostasis and mitochondrial homeostasis are interdependent. Mitochondria must import and incorporate iron ions to form iron–sulfur clusters, heme, and certain mitochondrial proteins that underpin cellular respiration (Figure 5) (118–120). While the deficiency in mitochondrial iron-sulfur cluster biosynthesis also triggered intracellular uptake and redistribution of iron (121). The absence of certain RNA-binding proteins in mitochondria can disrupt cellular iron homeostasis and lead to apoptosis (122). Therefore, the following sections elaborated on the association between mitochondria and ferroptosis in CVDs.

#### The role of ferroptosis in mitochondria

Mitochondrial morphology is an essential judge of cell death. They are generally short rod-shaped or spherical, 1–2 μm long, and consist of double membranes, an outer membrane and an inner membrane with a cristae structure. Ferroptosis is correlated with significant morphological alterations in the mitochondria. After the onset of the ferroptotic stimulus, fragmentation, condensed membrane density, lower volume, reduced or absent cristae and rupture of the outer membrane shortly occurs in the mitochondria (3, 123, 124). The tendency for reduction in mitochondrial membrane potential leads to mitochondrial dysfunction, as evidenced by increased ROS production, mitochondrial membrane hyperpolarization and mitochondrial swelling, ultimately leading to hypertrophy of CMs (113, 125–127). Both Fe^3+^ and Fe^2+^ overload can potentially cause mitochondrial disorders as described above, but Fe^2+^ has been proven to have more destructive effects than Fe^3+^ (128). Tadokoro et al. (129) revealed that excessive accumulation of free Fe^2+^ in mitochondria of CMs in mice with doxorubicin cardiomyopathy or I/R-induced HF, and that chelating Fe^2+^ in mitochondria prevented ferroptosis in CMs. Hence, mitochondrial damage due to Fe^2+^ overload was the main mechanism of cardiac injury (129). On the inner membrane of the mitochondria, the respiratory chain carries out electron transfer via four protein complexes (I, II, III, and IV) to produce ATP, which is the most basic energy metabolism in the organism. Chronic iron overload could additionally inflict damage on mitochondrial DNA and impair the synthesis of respiratory chain subunit complexes I and IV. These results explained chronic progressive cardiac hypertrophy and cardiac insufficiency (130). Treatment with the ferroptosis inhibitor liproxstatin-1 reduced myocardial infarcted size in mice by controlling the entry and exit of mitochondrial ROS via diminishing VDAC1, a pore-like protein in the outer mitochondrial membrane (131). Thus, ferroptosis accounts for impaired cardiac function by causing deterioration of mitochondrial function and interfering with mitochondrial dynamics.

#### The role of mitochondria in ferroptosis

Mitochondrial metabolism in the pathological state actively facilitates the generation of lipid ROS, and remarkably increases the level of loosely bound unstable iron ions, which is a prerequisite for ferroptosis (132). Mitochondria contains an abundance of GSH, accounting for about 10–15% of total intracellular GSH. Two carrier proteins, mitochondrial dicarboxylate and 2-oxoglutarate, have been identified as GSH transporters (133). Inhibition of these two carrier proteins resulted in the depletion of GSH and ultimately ferroptosis in CMs. In detail, when GSH was depleted rapidly, hyperpolarization of the mitochondrial membrane potential caused the release of cytochrome C and the activation of caspase, thus explaining the role of these two carriers in regulating ferroptosis (97, 134). In mitochondria, there are also organic compounds mitigating ferroptosis. Dihydrolipoic acid acts as an essential cofactor for several mitochondrial enzyme complexes, allowing the direct reduction of pro-ferroptotic peroxidized phospholipids to hydroxyphospholipids and scavenging oxygen radicals (135). It is speculated that more effective pharmacological interventions to protect mitochondria could be new therapeutic targets, improving clinical outcomes in the treatment of CVDs.

#### Mitophagy and ferroptosis

Mitophagy is a process that selectively scavenges damaged mitochondria and maintains normal mitochondrial activity. Specifically, the serine/threonine kinase PTEN-inducible kinase 1 (PINK1) and the E3 ubiquitin-protein ligase Parkin synergistically sense the functional state of mitochondria and label damaged mitochondria. The damaged mitochondria are encapsulated into autophagosomes and fused with lysosomes, thus completing mitochondrial degradation. Mitophagy was detected to protect against the development of HF, myocardial I/R injury, and obese cardiomyopathy through the Pink1/Parkin pathway (136–138). Mitophagy receptor deficiency can lead to cardiac remodeling and dysfunction through ACSL4-mediated activation of ferroptosis (134). However, overactivation of mitophagy may also induced HF by activating TLR4/NADPH oxidase 4 pathway (8), and mitophagy was also reported to trigger necroptosis and ferroptosis in melanoma cells (139). Therefore, mitophagy has diverse features under different conditions and the regulation of ferroptosis in CMs mediated by mitophagy may need to be further explored.

Several studies have come to the opposite conclusion that mitochondria are dispensable for the execution of ferroptosis induced by GPX4 inhibition, and mitochondria-deficient cell lines show no difference in the susceptibility of ferroptosis from control (3). Possible explanations are that firstly, once GPX4 was eliminated, small amounts of •O2–, •OH, and H_2_O_2_ will be quickly magnified by Fenton reaction, causing full-scale ferroptosis whatever the activity of mitochondria, and secondly, there may be some differences in metabolism between cell lines, with varying degree of dependence on mitochondria.

## Discussion and conclusion

Rapid advances in current evidence-based medicine for the treatment of CVDs firmly support the effectiveness of several drugs, such as angiotensin-converting enzyme inhibitors, beta-blockers and aldosterone receptor antagonists. Nevertheless, certain adverse side effect of these drugs has caused the administration restricted in specific patients. Recently, targeting iron homeostasis and ferroptosis has become a new direction for clinical treatment of CVDs. Two randomized clinical trials (FAIR-HF and Confirm-HF) have provided clear evidence for the benefit of iron supplementation in patients with chronic HF and iron deficiency. Although oral iron supplementation has no apparent effect, intravenous ferric carboxymaltose can improve hospitalization and mortality from cardiovascular events in iron-deficient HFrEF (LVEF ≤ 45%) patients (140). In terms of the secondary prevention of CVDs, a meta-analysis failed to identify participants more likely to derive clinical benefits from iron nutritional supplements in reducing the risk of cardiovascular outcomes (141). The benefit of iron supplementation in patients of acute CVDs without iron deficiency is unclear, and this treatment may exacerbate (I/R) injury. Conversely, administration of deferoxamine which chelates redox-active iron to inhibit ferroptosis has been shown to improve blood oxidative stress markers as an adjunctive therapy for patients with ST-segment elevation MI (142). Intravenous infusion of deferoxamine mesylate at the initiation of thrombolysis was also effective in ischemic stroke patients, with no apparent adverse effects (143). Therefore, pharmacological treatment to maintain iron homeostasis and inhibit ferroptosis may serve as a new therapeutic strategy for CVDs.

Additionally, although indicators of ferroptosis have predictive capacity on the prognosis of amyotrophic lateral sclerosis (144), ferroptosis biomarkers for predicting early diagnosis and prognosis of CVDs have not yet been reported clinically. Classic CVD-related ferroptosis biomarkers validated in animal models include GPX4, ROS, 4-HNE, COX-2, and iron levels, but there are still some limitations of detection (35, 45, 86). First, the activity of ferroptosis metabolites is time-sensitive and should be detected in time after the onset of CVDs. Second, oxidative stress also occurs during necrosis and apoptosis, and simple ROS detection cannot explain the occurrence of CM ferroptosis. Third, the great challenge for COX-2 as a biomarker of ferroptosis is that upregulation of COX-2 has been observed under a variety of inflammatory conditions, except in the case of myocardial ferroptosis. Finally, iron levels may not represent the level of ferroptosis and need to be measured in combination with other indicators. Other potential ferroptosis biomarkers associated with CVDs can be referred to Table 1. A recent bioinformatics analysis also determined elevated key genes associated with MI, ferroptosis, and hypoxia, such as Atf3, Socs3, Hspa1b, Cxcl2, and Myd88, which could serve as new biomarkers for MI (145). Thus, indicators of pathophysiology based on ferroptosis may have predictive potential for CVDs in the future, providing better stratification for patient personalized care and resource allocation.

In summary, ferroptosis-related pathological mechanisms such as oxidative stress, inflammation, metabolic disorders and mitochondrial damage are involved in the development of CVDs. As research proceeds, inhibition of ferroptosis may be an effective strategy for the treatment of CVDs.

## Author contributions

L-LZ and R-JT determined the topic, wrote the initial manuscript, searched the related literatures, and created the figures of the manuscript. Y-JY supervised the planning and execution of the research activity. All authors have approved to the final version of the manuscript, responsible for the accuracy, and authenticity of the article.
